# Assessment of progress in education for children and youth with disabilities in Afghanistan: A multilevel analysis of repeated cross-sectional surveys

**DOI:** 10.1371/journal.pone.0217677

**Published:** 2019-06-10

**Authors:** Jean-François Trani, Patrick Fowler, Parul Bakhshi, Praveen Kumar

**Affiliations:** 1 Brown School of Social Work, One Brookings Drive, Washington University in St. Louis, St Louis, MO, United States of America; 2 Program in Occupational Therapy, Washington University School of Medicine in St Louis, MO, United States of America; 3 School of Social Work, Boston College, McGuinn Hall, Chestnut Hill, MA, United States of America; Università degli Studi di Perugia, ITALY

## Abstract

Recent study shows that 617 million children and adolescents–or six out of 10 globally- are not acquiring minimum levels in literacy and mathematics, indicating the magnitude of the learning acquisition problem. For children with disabilities in context of conflict, the situation is arguably even worse: the literature shows that they face difficulties to access the education system due to multiple barriers, and when they do access, they are not learning. Our paper examines if an active education policy promoting inclusion since 2005 in Afghanistan, a protracted crisis context, has been effective. Using two cross sectional household surveys carried out eight years apart (2005–2013), our study shows that access to school and literacy did not improve between 2005 and 2013 for children and youth with disabilities. Both access and literacy outcomes were worse for girls with disabilities, those with a mental, learning or associated disability and those living in household where the head was uneducated. Finally, odds of being mentally distressed significantly declined between 2005 and 2013 indicating that schools might play a protective role for children with disabilities in Afghanistan. Our findings suggest that a multilevel multi-pronged adaptation of the existing system to improve the learning experience and promote children’s resilience, particularly for children with disabilities, in conflict context such as Afghanistan, is required.

## Introduction

Children who are most vulnerable to exclusion from, and marginalization within education, face many barriers to enrolling in and completing primary education particularly in Low Income Countries (LICs) [1]. When they do manage to enroll in schools, many vulnerable children do not learn at par with peers: 617 million children and adolescents–or six out of 10 globally- are not reaching minimum levels in literacy and mathematics [2]. Across the central and southern Asia region, it is predicted that 81% of children and adolescents (241 million) will not reach minimum proficiency in reading.

Despite existing provision in the United Nations Convention on the Rights of the Child (UNCRC, 1989) article 23,the UN Convention of the Right of Persons with Disabilities (UNCRPD, 2006) article 24 and the UN Sustainable Development Goal 4 (SDG4, 2015) referring, among other normative frameworks, to equal, free and quality education for all children, children with disabilities overall, and in LICs more specifically, are still particularly at risk of being out of school, or if enrolled, of not learning and reaching lower educational attainment that children without disabilities [3, 4]. Findings across 19 countries studied by Male and Wodon (2017) for the World Bank using census data show that the gap in school enrollment rate is 13.2 percentage points for boys and 12.7 points for girls, the gap in primary completion rates is 17.6 points for boys and 15.4 points for girls and finally the gap in literacy outcome is 16.2 points for boys and 15.5 points for girls between children with and without disabilities. One positive finding though is that girls with disabilities have closed the gap with boys with disabilities over the 47 years separating the youngest and oldest age groups studies by Male and Wodon. But overall, exclusion from education has a higher effect than any other factor such as gender, being an orphan, place of residence, household wealth or the level of education of the head of household, whatever the outcome considered. Such gaps can be considerably higher in some Low- and Middle-Income Countries (LMICs). For instance, the UN Flagship report on disability (2018) indicates that the gap in ever enrolling is 43 percentage points in Cambodia, 45 points in Indonesia, 38 points in Timor Leste and 35% in Vietnam between children with and without disabilities. Similarly, the report shows that the gap in completion rate is 17% in five LMICs (Cambodia, Colombia, Gambia, Maldives, Uganda) with the widest gap in Cambodia (29%) and Columbia (28%). Finally, persons 25 years and older without disabilities had 40% more time of schooling than persons with disabilities [4].

The situation is exacerbated in conflict contexts: violence and mistreatment in contexts of conflict cause deaths, disability, delay in development, anxiety and distress [5]. Conflicts strengthen various cycles that worsen overall vulnerability by increasing poverty, early marriage for girls and child labor, thereby preventing vulnerable children from accessing quality education. Children in conflict are often unable to go to school. In 2015 alone, 75 million children living in conflict zones did not have access to schools [6]. In Afghanistan specifically, respectively 40% or 2.3 millions of primary school age children–primarily girls–are out of school; mean years of schooling is 3.2 years [7]. Disadvantaged children, particularly children with disabilities, children in remote rural areas and from poor families, girls and ethnic minorities have lower enrolment rates and higher rates of repetition and drop out before completion [8]. As a result, children in conflict will lack essential skills and abilities to be the next generation of responsible citizens [9, 10]. Literature has also shown that conflict is a direct source of distress for children through the witnessing of violent events but also through the ongoing exposure to various daily stressors such as domestic or community violence and poverty [5, 11–14].

Inclusion of children with disabilities is particularly challenging in conflict settings: education systems are largely destroyed, newly reconstructed schools are not always accessible, reaching the school is a concern particularly for girls with disabilities because of risk of violence along the way and teachers lack adequate awareness and training to accommodate children with special needs [15]. Children with disabilities like other children are facing violence and daily stressors that take a toll on their mental wellbeing [16, 17]. Stigma of disability has been shown to increase even more mental distress in such contexts. Yet, there is some evidence that access to school and quality learning may provide a safe space in certain circumstances to promote inclusion, copying with trauma and support in navigating an unfriendly and violent environment that ultimately could improve child psychological wellbeing [18–20]. There is scarce evidence in the case of children with disabilities [17].

While studies have reported differences in school access and attainment between children and youth with and without disabilities in LICs at a given time, including in conflict settings [17], to our knowledge, none has empirically examined the impact of investing in primary education on the achievement for children with disabilities specifically. Furthermore, the potential protective role of school availability against anxiety and distress in a protracted crisis or conflict context has not been assessed for children with disabilities. The scope of the present study was therefore to explore progress made in including children with disabilities in the classroom, improving their basic learning outcomes and protecting their emotional and psychological wellbeing following the general investment made in the education system.

The present study investigates the following research questions:

1. Is investment in education associated with better school access for children with disabilities?
2. Is investment in education linked to better learning outcomes; and
3. lower mental distress?

Following this introduction, the second section -background- describes the notion of quality education for all and its implementation in conflict settings, specifically in the context of Afghanistan. Section three details the methods, study design and sampling and measurement of access to school, learning outcomes and psychological wellbeing. Section four provides results related to our outcomes of interest. Finally, section five discusses findings and concludes.

## Background

### Quality education for all: A challenging yet crucial goal in conflict contexts

The Education for All framework focused on quality and equity in education [21, 22]. Yet, the UN’s Millennium Development Goal 2 aimed at universal primary education, focusing primarily on indicators of school enrolment and excluding the measure of cognitive and non cognitive learning achievements. Although the net enrolment rate increased from 83% in 2000 to 91% in 2015, data shows that 57 million school-age children are still not in schools and a considerable amount are not learning in schools in low-income contexts, particularly in protracted crisis contexts, such as in Afghanistan [23–26]. The need to shift focus towards inclusion and quality education is outlined in the Sustainable Development Goal 4 (SDG4) including for countries in conflict or crisis contexts [27, 28]. This goal of achieving universal, quality and free primary education by 2030 will only be achieved if children with disabilities are included in the classroom and benefit from full and equitable participation in the education system with the appropriate support, as stated in article 24 of the United Nations Convention for the Rights of Persons with Disabilities (UNCRPD) [29].

Many of the impediments faced by children with disabilities in conflicts are constraints faced by all children [15]. Education does not systematically constitute a priority for donors or governments [30]. The education system is characterized by lack of facilities, overcrowded classrooms and paucity of trained teachers that hinder quality. Exogenous factors include security concerns, cost of education for families and contribution to farming, other work or household chores. Furthermore, national security constitutes a priority over education budgets, which result in low pay for teachers, poor infrastructure, limited resources. This in turn reduces quality of education received. Children with disabilities face additional and specific challenges due to the circumstances relating to their impairment. First, there is limited provision of guidelines to include children with disabilities in the education reconstruction process. However, the Inter-agency Network on Education in Emergencies (INEE) does mention accessibility of buildings, promoting awareness of various stakeholders (children, parents, teachers, humanitarian workers and policy makers), training and capacity building of teachers and use of local resources [31]. Second, there is a considerable knowledge gap about how to promote inclusion of children with disabilities in conflict settings [32]. Third, resources are scarce and often insufficient to achieve goals of universal quality education [33]. Finally, existing beliefs such as the idea that children with disabilities need special schools to learn and cannot be included in mainstreaming schools must be overcome [4].

Yet, the literature has shown that building an inclusive education system after war constitutes a unique opportunity to promote economic, social and political change and particularly foster social justice [34]. Five roles have been identified to comprehend how education contributes to peacebuilding [35]. First, to promote peace and reconciliation, since the 1980’s the international community has promoted programs of Disarmament, Demobilization, and Reintegration (DDR) that include skills training to help economic reintegration of ex-combatants, including child soldiers [36]. Unfortunately, multiple studies have shown that reintegration can be threatened by numerous elements such as lack of reconciliation [37], little consideration for local contexts and needs of program recipients [38] and poor participation of recipients in the definition and implementation of the program [39]. Second, it has been argued that education can offer a protective environment for children particularly through emotional, psychological and cognitive development [40, 41] while protecting them from forced recruitments, exploitation and prostitution [6, 42]. Third, education can provide a sense of restored normality for children through the construction of schools and the reintroduction of a routine of learning [43]. Fourth, an inclusive education system might rectify previous injustice, help society to recover from conflict, raise awareness about the opportunity cost of engaging into violence and make simple messages of charismatic leaders less appealing to educated citizens [34, 44–46]. Fifth, child education is the condition of future economic development, better child health outcomes, peace and security, and paves the way for good governance as well as active and engaged citizenship [35, 44, 47].

### Threats to quality education for all in Afghanistan

National Afghan policies also promote inclusion and quality education. In post-Taliban Afghanistan, access to learning has been recognized as a strong means towards sustaining development and building peace [48]. As a consequence, considerable effort has been made to address education needs. Between 2001 and 2013, 14600 schools have been newly established -of which 6100 are primary schools and 187,000 teachers have been recruited; 70% of these are primary level teachers [49]. Today, it is estimated that there are 215,000 teachers in Afghanistan of which 20% are women. Furthermore, textbooks and teachers guide have been provided as well as essential commodities in particular water and sanitation. The Ministry of Education (MoE) states that 8.3 million of the 10.3 million school-age children are now in schools [50]. Policy papers of the MoE have repeatedly emphasized the substantial challenge that inclusion of all children, particularly children with disabilities, those from poor households, returnees and displaced children constitute for the education system [8].

Among new initiatives, and in order to promote access to school in remote areas, the MoE has developed community-based education at the village level, increased access to training for female teachers and sensitized communities to promote girls’ school-enrollment despite religious and traditional beliefs [51]. These efforts reduced the gender gap in school enrollment and in basic learning test scores but were found to be very sensitive to the distance to school [52]. Village-based schools offer an essential opportunity to improve primary education in rural areas of Afghanistan, and the need to work closely with the local councils (*Shuras*) has become evident in order to achieve equity. Some initiatives have been attempted to empower marginalized groups such as the BRAC’s life skills education and livelihoods trainings for young Afghan women [53].

Despite considerable progress in increasing the number of schools, teachers and students’ enrollment, prioritizing children vulnerable to exclusion: 1) girls; 2) children living in remote rural interiors; 3) and those with disabilities, have been a challenge. This has led to in the past to inequitable access to education, and poor learning experience with lower educational outcomes for those enrolled. [32, 54–56]. Although UNESCO has developed a toolkit and other material to address inclusion and quality in classrooms in Afghanistan [57], its operationalization in primary schools and by NGOs might have been limited due to the absence of an effective and concerted strategy. The inclusion of vulnerable children in the Afghan education system with an increased focus on quality education is further endangered by widespread practices of corruption and nepotism left unchecked due to the absence of close and reliable monitoring of the education sector’s performance by donor agencies [58]. Corruption cripples investment made in the education system at multiple levels: recruitment, school management, teaching practices and overall performance of the system. In absence of transparency in recruitment, the 2017 study carried out by the Independent Joint Anti-Corruption Monitoring and Evaluation Committee (MEC) indicates that newly graduated teachers from the Teacher Training Colleges (TTCs) are often required to pay a bribe between AFN 50,000 and 70,000 ($800 to $1000), worth more than a year of wage. Numerous trained teachers cannot afford to pay such an amount for a formal teaching position while unqualified individuals are appointed instead. Another consequence of this system of bribery is that many trained women cannot obtain teaching positions. This situation is particularly preoccupying considering that the overall Gender Parity Index (GPI) is of 0.66 (6.6 women teachers for 10 men) and can be as low as 0.1 in provinces such as Zabul or Uruzgan preventing girls from being educated since many families would not allow their daughters to go to schools where teachers are male. Finally, corruption threatens quality teaching by encouraging teacher absenteeism, shortened school sessions, non-distribution of government-issued books resold on the black market and little engagement in class with students.

### Education investment using available data

Using two cross sectional household surveys carried in the provinces of Afghanistan in 2005 and 2013, we investigate the effect of investing in education in Afghanistan on effective inclusion in education of children with disabilities. To the best of our knowledge, the National Disability Survey in Afghanistan (NDSA, 2005) and the Disability Program Impact Evaluation Study (DPIES, 2013) are the only available randomly selected household-based surveys screening for disability using the same validated instrument and investigating education in Afghanistan across the same 13 provinces (out of 34). We hypothesize that investment in the education system translates into increased access, improved learning process and stronger protective mechanisms for the mental wellbeing of all children with disabilities. We are testing if the investment in infrastructure by the state and the international community, resulting in an increase in the number of schools and teachers, together with a better connection of the villages to the outside world by road and through access to electricity (allowing use of mobile phone, and receiving television and radio programs) between 2005 and 2013 have a positive impact in terms of student access, learning achievement and protection of their mental wellbeing.

## Methods

### Study design and setting

We carried out two household surveys in 2005 and 2013 using a similar method: In 2005, we followed a three-stage clustered randomized sample design [59]. At the first stage, we randomly selected 121 districts (out of 397) within the 34 provinces of Afghanistan using a proportional to size method. At the second stage, 175 clusters were randomly selected within those districts. Finally, within selected clusters, 30 households were randomly selected. We rolled a pointer from the center of the village and numbered 30 households in the random direction identified [59]. We then selected the first household by picking randomly a number between 1 and 30 and selected the other 29 household using the nearest front door method. All household heads were interviewed about household composition and each member demographic and socioeconomic characteristics. We also interviewed the head of household with a 27-item disability screening tool locally developed and validated to identify all members of the family with disabilities. For the present study, we examined education outcomes for children and youth with disabilities between 6 and 25 years old in the same 13 provinces surveyed again in 2013. We therefore excluded the data for the other 21 provinces not surveyed in 2013. We limited the observation to children and young adults because very few adults accessed education when they were children in the period preceding 2001 dominated by the Taliban regime.

**Fig 1:**
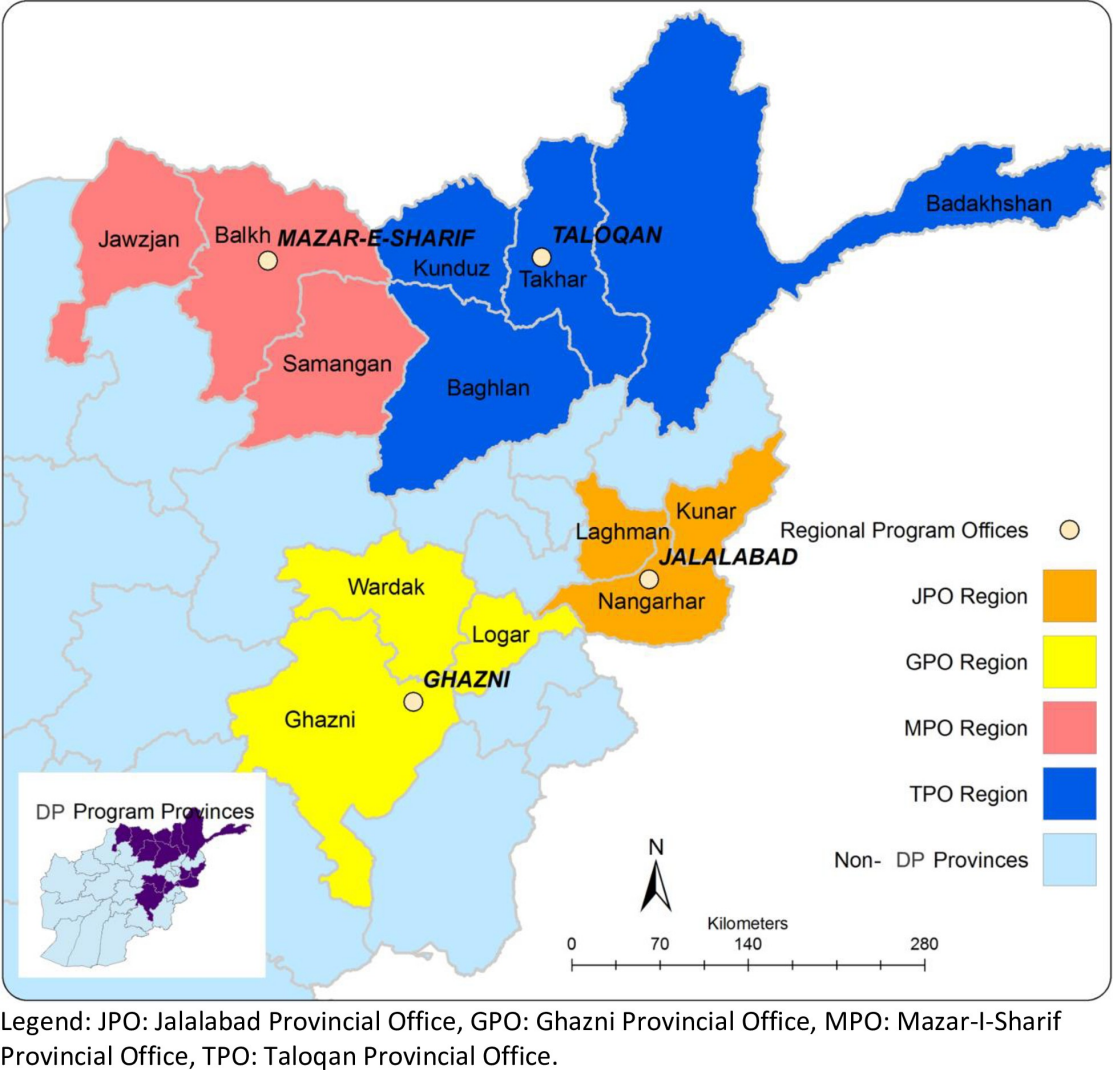
Map of Afghanistan regions and provinces representatively sampled in 2005 and 2013.

In 2013, we revisited the same 13 provinces out of the 34 provinces assessed in 2005. The provinces were purposively selected based on whether a home-based disability program was implemented. These 13 provinces in the Northeastern region of Afghanistan (see Fig 1) were receiving better education support from NGOs in 2013 than some other provinces of the country where the presence of armed opposition groups was reducing access to school for children, particularly girls in Taliban controlled areas. Therefore, we are less confident that other provinces have seen as much investment in education as the 13 provinces where the DPIE is implemented.

**Fig 2:**
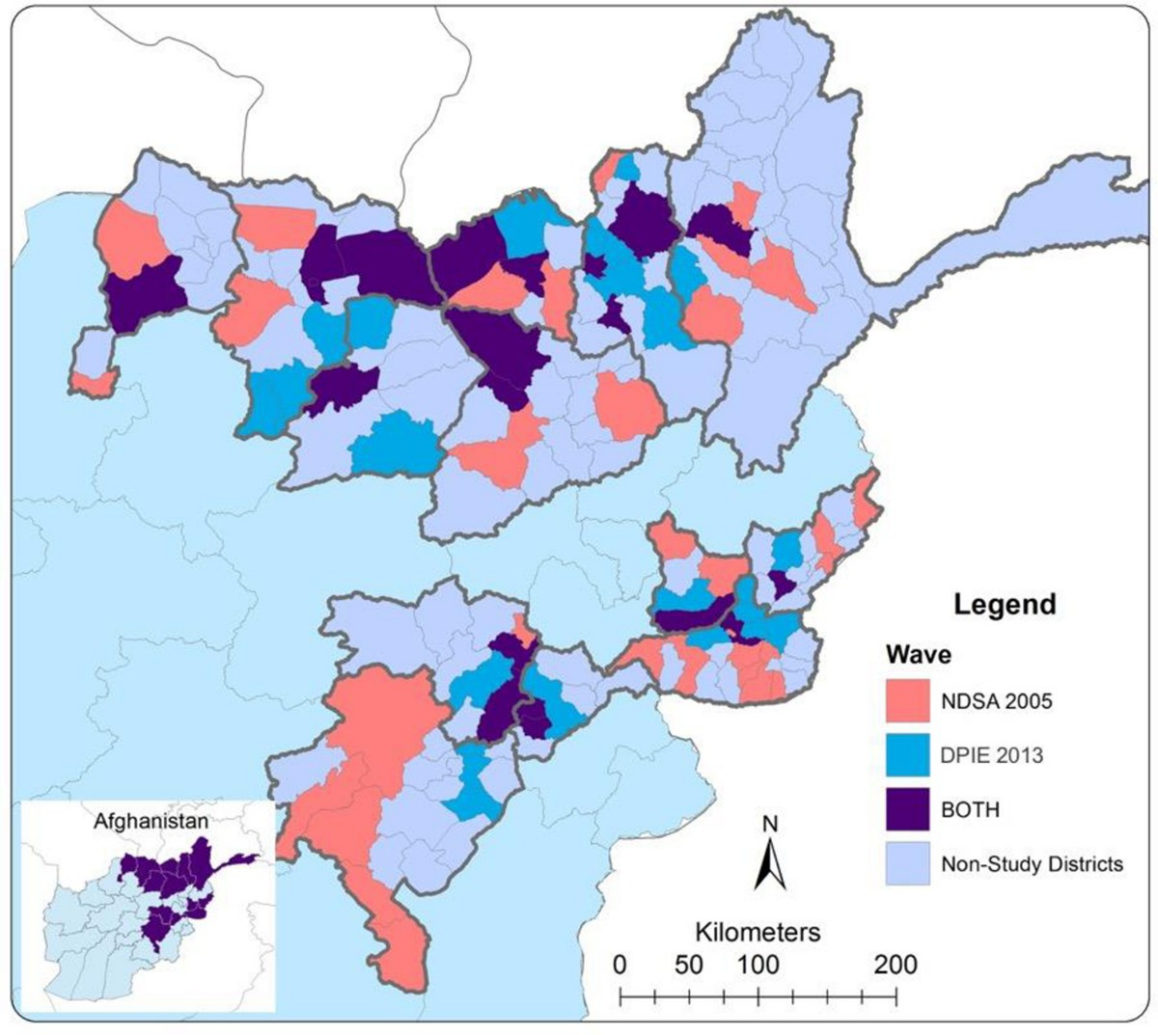
Map of Afghanistan districts sampled in 2005 and 2013 household surveys.

Within the 13 provinces, in districts located outside of the catchment area of the home-based disability program, households were identified using a two-stage randomized cluster sampling technique with villages (N = 107) as primary sample units within the same provinces and their districts (administrative subdivisions of provinces). Out of total of 76 districts selected within those 13 provinces, 20 (26.3%) were part of both surveys, 32 (42.1%) were part of the NDSA only and 24 (31.6%) were part of the DPIE only (Fig 2).

In each village, 60 households were randomly surveyed and the head of household was interviewed to identify members of the household having a disability using a 34-item updated version of the disability screening tool [60]. For the purpose of the present study, only children and youth between 6 to 25 years old screened as having a disability were interviewed with a similar locally developed and validated questionnaire asking about education and emotional status among other topics. Both samples represent Afghan families with children who have disabilities.

Taliban occupation did not excessively disrupt study procedures. In most villages, the team was welcomed to conduct the survey. We had to remove a few villages from the sampling frame where Taliban occupation did not allow for a survey. Interviews with children and youth with disabilities were carried out by a team of enumerators locally recruited and trained after providing written or verbal consent. In both surveys, caregivers were present for the interviews of children. Children and youth with disabilities were interviewed using another tool about education and emotional status and several other themes. Instruments were carefully developed in consultation with local experts in child disability and piloted among Afghan families with children who have disabilities. Enumerators in 2005 and 2013 were trained to explain the questions and provide examples when needed. The 2005 study received ethical approval from the Committee on Human Research of the Johns Hopkins Bloomberg School of Public Health and the Ministry of Public Health of Afghanistan and the current study received approval from The Human Research Protection Office at Washington University in St. Louis and the Ministry of Public Health of Afghanistan.

Table 1 summarizes outcome and predictor variables. We will first discuss the three outcome variables followed by the predictor variables.

**Table 1:** List of outcome and predictor variables.

Outcome variables				
Access to school	At least one year of education			
Literacy	Able or not to read and write a short sentence			
Mental distress	Either anxious or sad			
Predictor variables				
Level 1				
Age	Years			
Gender	Female or male			
Type of disability	Physical, sensory or Mental, learning and associated			
Cause of disability	By birth or acquired after birth			
Size of the family	Number of members of family living in the household			
Ethnicity	Pashtun, Tajik or minority			
Head of household access to school	Educated or no education			
Head of household employment status	Working or not			
Asset index	20% poorest, middle 20%-80%, 20% richest			
Level 2				
Electricity in the village	Yes or no			
Village connectivity by a paved road	Yes or no			
Presence of a school in the village	Yes or no			
Year	2004/5 and 2013/14			

### Outcome variables

#### Access to school

To analyze access to school, both surveys used a self-reported measure of whether children attended school or not. We considered a minimum of one year of enrolment, which is routinely accepted as indicator for access to school [61].

#### Literacy

At the time of our study, no standardized test for basic reading and writing such as the Monitoring Education Development in Afghanistan (MED-A) framework developed by the Australian Council for Educational Research in Dari and Pashto were available. Therefore, basic learning cognitive skills were evaluated through literacy assessments that asked children to both read and write a short sentence used in both surveys: “My name is _____. I can read and write”. The enumerator would probe the child to assess reading and writing skills by asking to write eventually more: “my village name is___” until she or he would have a better idea of the literacy skills of the child respondent.

#### Mental distress

The outcome, mental distress was measured using two locally tested and validated questions about feelings of anger and sadness in 2005 and 2013. We could not identify one single measure easily adaptable to the cultural context. Literature has argued that mental disorders are not always well identified using Western developed measures. In Afghanistan, it has been shown for instance, that the Hopkins Symptom Checklist-25 (HSCL-25) and the Self-Reporting Questionnaire-20 (SRQ-20) had relatively poor properties to correctly identify mental disorders [62]. Therefore, we developed, tested and validated our own measure. In the 2005 study, anger and sadness was defined using two “yes” or “no” questions asked to children: “Do you become very sad/cry without reason?”; “Do you feel angry and resentful for no particular reason?”. In the 2013 study, mental distress was assessed by items that asked “Do you feel sad?” and “Do you feel angry?” with three possible responses for each question: “No, I never feel [respectively] sad/angry” or “Yes, I sometimes feel [respectively] sad/angry” or “Yes, I always feel [respectively] sad/angry”. We retained sadness and anger as indicators of mental distress because of the recurrence of these two items in focus group discussions and semi-directive interviews and because they were also identified by the Afghan Symptom Checklist [63, 64].

Assessment for mental distress was further tested for accuracy, completeness and content validity. We conducted a series of individual interviews (with a sample of children and youth with disabilities), their caregivers, and consulted with Afghan medical and rehabilitation experts for cognitive response testing (CRT). CRT is routinely used in refining a measure, to improve the quality of data collection, and to improve the validity of the response. CRT determines: 1) question comprehension (e.g., What do specific words or phrases in the question mean to the respondent?); 2) information retrieval (e.g., What information does the respondent need to recall in order to answer the question?); and 3) decision processing (e.g., How do they choose their answer?). We tested if the questions adequately covered the underlying concept of anger and sadness in Dari, and Pashto. CRT results indicated that they were robust proxies of overall mental distress [65, 66]. Respondents confirmed that sadness and anger reflected a state of mental suffering that has been associated in Afghanistan and other conflict affected settings with continuing violence, chronic poverty and social exclusion [5, 67, 68]. A dichotomized indicator assessed whether children felt any sadness versus no sadness or anger.

### Predictor variables

The multilevel model measured individual and community-level characteristics (Table 1). Individual level variables were gender, age, ethnicity, cause and type of disability, education and employment status of the head of household, family size and asset index. The asset index is based on a principal-components analysis of 12 major assets and by deriving the asset tertiles from the first factor of the analysis [69]. Community level predictors included measures of village remoteness. This included whether the villages had electricity, were connected by a paved road, and whether villages had a school. We hypothesize that after controlling for socio-economic and demographic variables at level 1 and level 2, investment in education will have a positive impact on: 1) access to school; 2) literacy; and 3) mental wellbeing of children and youth with disabilities in 2013 compared to that in 2005. We measure investment in education by “presence of a school in the village”, which is a level 2 variable in our model.

### Statistical analysis

The repeated cross-sectional research design with stratified random selection sample provided strengths and weaknesses for testing study questions. Data provided the most rigorous and representative source for gauging the wellbeing of children with disabilities across Afghanistan. Moreover, assessments occurred before and after a highly volatile period that reshaped the political landscape, and thus, gave a unique opportunity to examining associations between educational investments and child outcomes. At the same time, the design suffered from the inherent challenges of using observational data to probe causal inference [70–72]. In the absence of randomization at the child-, household-, or village-level, stratified random selection fails to isolate causal effects of educational investments. Moreover, counterfactual approaches that leverage observational data remain prone to bias associated with unobserved confounds (ignorability assumption) and household residential mobility across villages (stable unit treatment value assumption) [73, 74]. Hypotheses that test village effects on child outcomes further challenges counterfactual estimation given the need for observations of potential outcomes at multiple levels [74–76]. Analyses required a flexible approach that maximized the accuracy of information for answering the important study questions.

**Fig 3:**
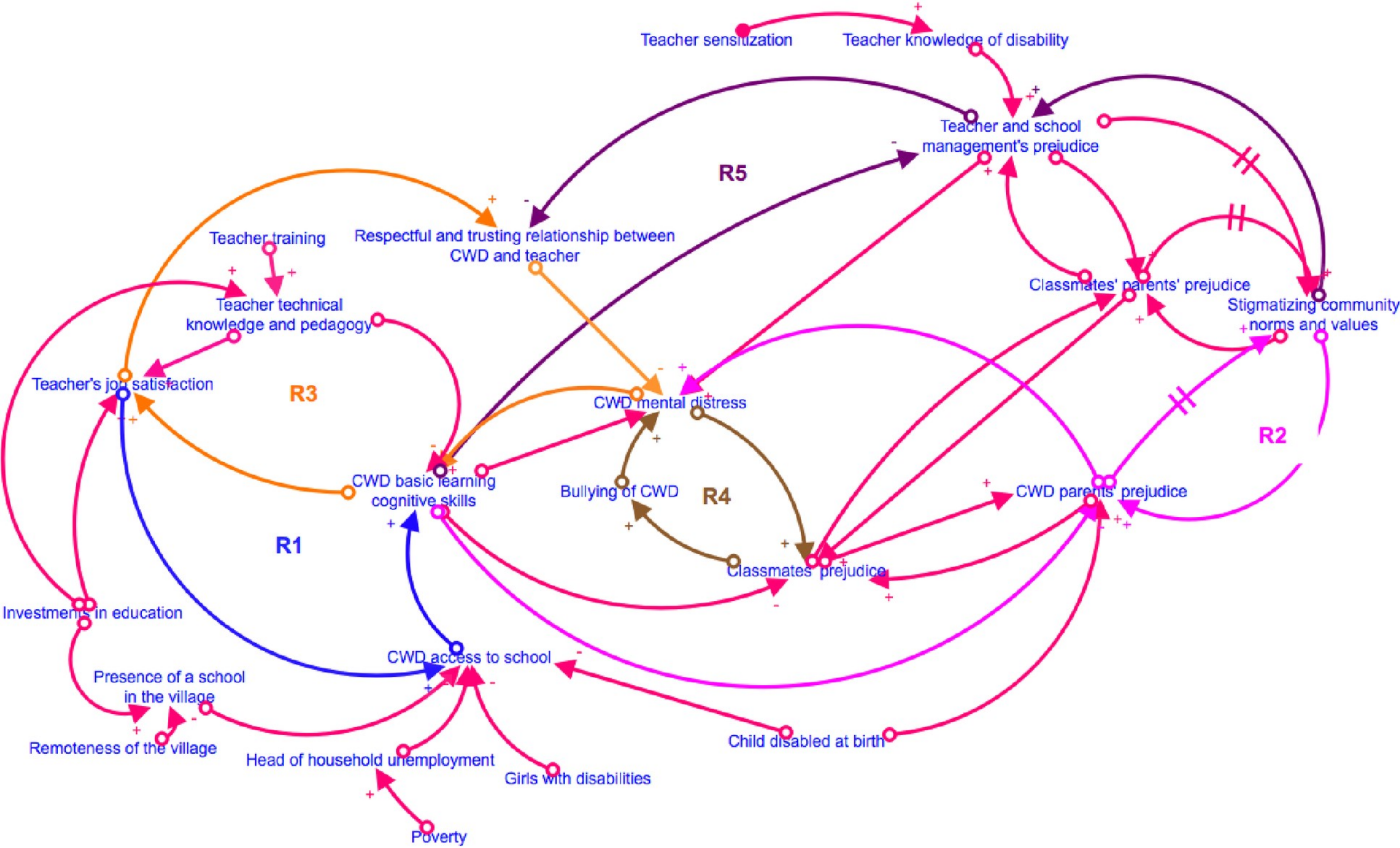
Causal loop diagram of investments in education and children with disabilities access to school, learning and mental wellbeing.

Multilevel modeling used a theory-driven approach to test hypotheses [70, 77, 78]. A theoretical graphical model or “causal loop diagram” (see Fig 3) visually articulates the key variables and associations between variables assumed to explain how investments in education influence the learning outcomes and mental distress of children with disabilities in Afghanistan [79]. The model was used to identify potential confounds; factors associated with child outcomes as well as whether children were able to access educational investments (e.g., gender, type of disability, household poverty). Arrows, or links, represent causal relationships. The plus and minus symbols of the model indicate the polarity, or the direction of the causal relationship. The plus sign indicates a relationship that goes in the same direction, a minus sign represents an inverse relationship. For instance, the more children are disabled at birth, the less access to school. Another example would be: The more remote is the village -far away from a paved road and deprived of electricity- the less likely to have a school in the village. Reinforcing loops indicate some of the relationships between factors. “R1” for instance shows that more teachers are satisfied with their job, the more they will be keen to accept children with disabilities, the more these children will learn, the more teachers will be satisfied with their job. Investments in education influence “R1” through both job satisfaction and by allowing more schools to be built. Although data collection failed to capture the full theoretical process, observations were available for a certain number of key factors. A few key variables that are not measured in our models are teacher job satisfaction, and school community stakeholders’ (parents, children, teachers, school management) prejudice towards disability. For this reason, we must consider with caution any attempt at understanding our models as providing causal estimates between investments in education and children with disabilities’ access to school, basic learning cognitive skills and mental wellbeing.

We conducted multilevel logistic regression analyses with random intercepts to test our hypotheses. Dependent variables were binary indicators of: 1) child school access; 2) literacy; and 3) mental distress [80]. Level 1 regressed outcomes on individual and household characteristics, while level 2 regressed the random intercepts on village characteristics, including the year of assessment. Between 2005 and 2013, broad educational investments were made by the Government of Afghanistan such as construction and equipment of schools, training of teachers. We tested our hypotheses to explore improvement in outcomes (access to school, literacy, and mental wellbeing) by investment in education in 2013 relative to 2005 after accounting for household and village effects on school access, literacy, and mental distress. Covariates were centered at the village mean, and random intercepts allowed effects to vary across villages.

Multilevel models regressed each outcome (access to education, literacy, and mental wellbeing) on covariates simultaneously. The multivariate regression analyses fit conceptualization that predictors influence multiple outcomes. In addition, incorporation of multiple outcomes allowed the use of full information maximum likelihood to handle missing outcome data (school access < 1%, literacy = 11%, mental distress < 1%) [80–82]. Analyses used a maximum likelihood estimator with robust standard errors and numerical integration. The log-likelihood, Akaike information criteria (*AIC*), and the Bayesian information criteria (*BIC*) were used to assess model fit [83]. We considered a p value of 0.05 to be statistically significant and used Mplus statistical software version 8.0 for all analyses.

## Results

### Sample characteristics

Table 2 shows key socio-demographic features in the representative samples of Afghan families with children experiencing disabilities. Characteristics of randomly selected villages varied considerably between 2005 and 2013 as a result of the significant economic investments across the country. In 2005, most villages did not have electricity or schools, and only half were connected by paved roads to other parts the country. By 2013, the remoteness of randomly selected villages reversed so that most had electricity, location of the closest school was nearer, and distance to paved roads were overall considerably reduced.

**Table 2:** Distribution of outcome and predictor variables by cohorts.

Variables	NDSA 2005 (N = 163)	DPIE 2013 (N = 537)
Village predictors		
Electricity in the village Yes	12.30%	76.70%
No	87.70%	23.30%
Village connectivity by a paved road Yes	42.90%	82.50%
No	57.10%	17.50%
School in the village Yes	28.80%	92.70%
No	71.20%	7.30%
Individual predictors		
Gender Female	43.60%	38.20%
Male	56.40%	61.80%
Age Mean (SD)	13.7 (6.0)	14.5 (6.3)
Ethnicity Pashtun	46.00%	37.50%
Tajik	28.20%	34.40%
Minority	25.80%	28.10%
Type of disability. Physical	39.90%	48.90%
Sensory	26.40%	22.20%
Mental and associated	33.70%	28.90%
Cause of disability By birth	61.40%	44.90%
Acquired after birth	38.70%	55.10%
Asset index 20% poorest	17.70%	20.50%
20%-80%	64.60%	57.50%
20% richest	17.70%	22.00%
Head of household primary educated Yes	36.80%	22.80%
No	63.20%	77.20%
Head of household working Yes	88.30%	79.60%
No	11.70%	20.40%
Size of families Mean (SD)	9.1 (3.6)	10.3 (7.5)
Outcome variables		
Access to school Yes	32.50%	27.50%
No	67.60%	72.70%
Read and write Yes	20.30%	15.60%
No	79.80%	84.40%
Mental distress Yes	36.80%	30.50%
No	63.20%	69.50%

Note: NDSA 2005: National Disability Survey in Afghanistan 2004/05; DPIE 2013: Disability Program Impact Evaluation Study, 20013.

The individual and household characteristics of families with children with disabilities had overall the same distribution as the overall Afghan population. Children with disabilities were more likely to be male and were in early adolescence years. Youth were primarily Pashtun, the largest ethnic group in Afghanistan that comprises approximately two-fifths of the population. Children with disabilities from the second largest ethnic group Tajiks comprised approximately 30% in both surveys, while the remainder of children came from the various smaller ethnic minority groups, such as Hazara, Uzbek, and Aimak. Youth most commonly experienced mobility limitations or physical disabilities as the primary impairment in both years with mental disabilities comprising approximately one-third and sensory problems the remaining one-quarter of youth. Approximately half of the children had disabilities at birth versus acquired during childhood. The majority of households were headed by employed but uneducated fathers providing for nearly 10 people in the family. None of the household characteristics varied when aggregated to the village level. Subsequent analyses allowed the effects of household characteristics on child outcomes to vary within villages to account for any individual-level differences by year.

### Outcome analyses

Table 2 shows the frequency of child outcomes by year. Less than one-third of children with disabilities attended school. Access declined in 2013 despite significant increases in the proportion of villages with schools. Similarly, literacy rates decreased to less than one in six children in 2013. The proportion of children with disabilities experiencing mental distress reduced slightly from almost two in five in 2005 to 30.5% in 2013.

Initial two-level models regressed child outcomes on household predictors nested within villages without including observed village characteristics. This unconstrained model indicated significant shared variance in child outcomes associated with village residence. The model provided adequate fit to the data (*LL* = -730.49, *AIC* = 1544.92, *BIC* = 1720.83). Intra-class correlation (ICC) coefficients estimated that villages accounted for 28% of the variability in school access, 14% literacy rates, and 25% of child mental distress. The unconstrained models demonstrated the importance of villages in explaining child outcomes, as well as the nested structure of the data [83].

Table 3 presents parameter estimates from the final model that regressed child outcomes on within and between village characteristics. Inclusion of the observed village characteristics improved model fit (*LL* = -717.92, *AIC* = 1543.83, *BIC* = 1770.00; Δχ^2^ (12) = 25.08, *p <* .05). Estimates of R-square by level suggested household characteristics explained significant variance in school access (27%) and literacy (32%) but not mental distress (9%), whereas village characteristics explained significant portions of mental distress (35%) and not school access (14%) or literacy (10%). Thus, individual household characteristics were more closely associated with educational outcomes, whereas village residence accounted more for child mental distress.

**Table 3:** Multilevel logistic regression analyses for access to school, literacy and mental distress for children with disabilities in Afghanistan.

Predictor variables	Access to school				Literate or not				Mental Distress			
Level 1	OR	95% CI		P value	OR	95% CI		P value	OR	95% CI		P value
Age (years)	0.935**	-0.091	-0.045	0.004	1.042**	0.02	0.062	0.052	0.928***	-0.096	-0.054	<0.001
Gender (ref: Male)												
Female	0.366***	-1.262	-0.748	<0.001	0.468*	-1.071	-0.445	0.015	0.794	-0.488	0.026	0.369
Type of disability (Ref: Mental and associated disability)											
Physical	3.219***	0.839	1.499	<0.001	9.608***	1.748	2.778	<0.001	1.576	0.134	0.776	0.156
Sensory	1.633	0.085	0.895	0.226	3.183	0.555	1.761	0.055	1.397	-0.059	0.727	0.393
Cause of disability (Ref: By birth)												
Acquired after birth	3.446***	0.953	1.521	<0.001	1.443	0.064	0.67	0.226	0.755	-0.531	-0.031	0.261
Size of the family	1.015	0	0.03	0.328	1.013	-0.006	0.032	0.504	1.015	0.002	0.028	0.257
Ethnicity: (Ref: Minority)												
Pashtun	0.681	-0.699	-0.069	0.224	0.73	-0.722	0.094	0.441	1.09	-0.29	0.462	0.821
Tajik	0.997	-0.368	0.362	0.995	1.257	-0.184	0.642	0.58	0.795	-0.575	0.115	0.505
Head of household access to school (Ref.: No access)	1.785*	0.301	0.857	0.037	1.834*	0.321	0.891	0.033	0.687	-0.65	-0.1	0.172
Head of household access to work (Ref.: Not working)	1.575	0.155	0.753	0.129	1.53	0.02	0.832	0.295	0.967	-0.394	0.328	0.929
Asset index: (Ref: 20% richest)												
20% poorest	0.562	-1.056	-0.096	0.231	1.102	-0.417	0.611	0.85	0.796	-0.777	0.321	0.677
20%-80%	0.733	-0.643	0.021	0.35	0.801	-0.55	0.106	0.5	0.837	-0.48	0.124	0.554
Level 2	β	SE	Est./S.E.	P value	β	SE	Est./S.E.	P value	β	SE	Est./S.E.	P value
Electricity in the village (Ref.: No)	-0.335	0.519	-0.646	0.518	-0.105	0.543	-0.193	0.847	-0.254	0.277	-0.915	0.360
Connectivity by a paved road (Ref: No)	0.492	0.475	1.035	0.301	0.179	0.470	0.381	0.703	-0.119	0.284	-0.418	0.676
Presence of a school in the village (Ref.: No)	0.453	0.595	0.762	0.446	0.463	0.629	0.736	0.461	-0.279	0.328	-0.849	0.396
Year (Ref.: 2005)	-0.741	0.622	-1.191	0.234	-0.817	0.642	-1.273	0.203	-0.807*	0.340	-2.374	0.018
Log Likelihood (LL)	-717.92 1543.83 1770.0											
Akaike information criteria (AIC)												
Bayesian information criteria (BIC)												

Note: OR = odds ratios; 95%CI = 95% coefficient interval, β = unstandardized betas; SE = Standard errors; Est./S.E = parameter estimate/standard error; significance at the

***(p < 0.001)

**(p < 0.01)

*(p < 0.05).

Results provided only partial support for study hypotheses:

#### Access to school

Afghan children with disabilities surveyed in 2005 and 2013 reported no significant differences on school access (*b* = -.74, *SE* = .62, *p* = .23) after accounting for household level characteristics. Girls with disabilities were respectively 2.7 times less likely to access school than boys with disabilities. Similarly, the odds of accessing school were 3.2 times higher for children with physical disability compared to children with mental and associated disabilities.

#### Literacy

Children with disabilities surveyed in 2005 and 2013 also reported no significant differences in literacy (*b* = -.82, *SE* = .64, *p* = .20) after accounting for household level characteristics. Girls with disabilities were 2.1 times less likely to be literate than boys with disabilities. The odds were 9.6 time higher for children with physical disabilities compared to children with mental and associated disabilities.

#### Mental distress

**Fig 4:**
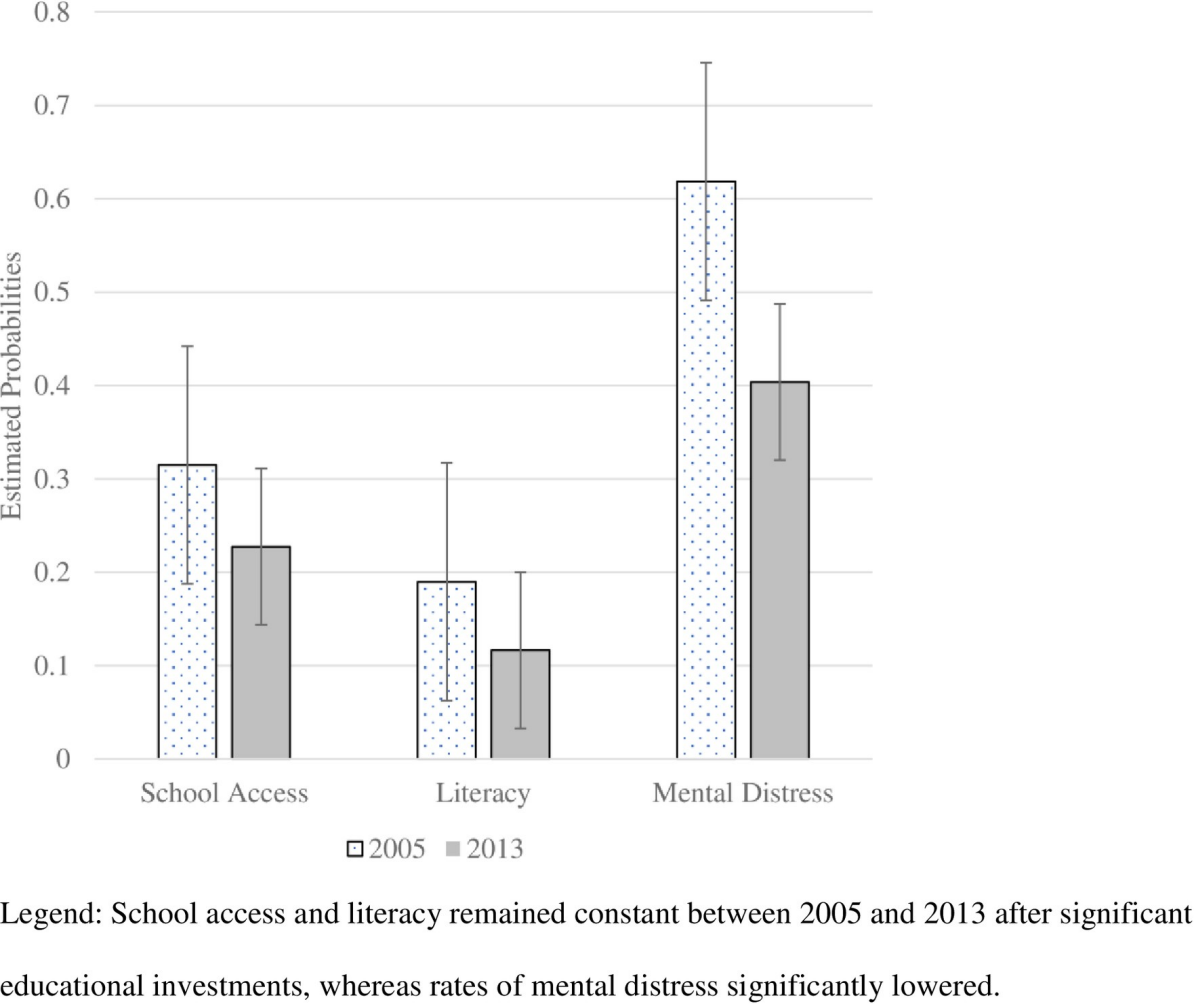
Estimated probabilities of educational and emotional outcomes for Afghani children with disabilities in 2005 and 2013.

However, children with disabilities in villages surveyed in 2013 reported significantly lower mental distress compared to those surveyed in 2005 (*b* = -.81, *SE* = .34, *p* = .02), which means child mental distress decreased by almost one standard deviation. No other village characteristics predicted literacy, school access, nor mental distress. Fig 4 details estimated probabilities of child outcomes by survey year.

A number of household-level covariates significantly predicted child outcomes as displayed in Table 3. Findings of the model predicting access to school and literacy in Afghanistan for children and youth with disabilities were worse for girls with disabilities, those with a mental, learning or associated disability and those living in households where the head was uneducated. Disability acquired after birth was positively and significantly associated with access to school but not literacy. The odds of children disabled after birth or from a known cause to go to school were 3.9 times higher than those of children born disabled but the odds of becoming literate were not significantly different according to the cause of disability. Older children and youth were less likely to be distressed and anxious than younger children. The fact that there was a school in the village did not influence the probability of being mentally distressed for children with disabilities.

## Discussion

Our study examined if investment in the education system has led to better access to school, literacy and protection against anxiety and distress in Afghanistan for one of the most vulnerable groups often excluded from school, children and youth with disabilities. This study constitutes an important contribution to the literature because there is little evidence about educational achievement of children with disabilities in crisis contexts, particularly in relation to improving mental and emotional wellbeing [84]. When literature does exist, they usually comprise of observational studies at a single point in time [84].

Our findings show that mental distress of children with disabilities reduced during the period considered (2005–2013). But, improvement in children with disabilities’ mental wellbeing cannot be directly attributed to the presence of a school in the village. Conversely, access to school and literacy rates did not improve for children and youth with disabilities between 2005 and 2013. Even after considering investment such as creation of a school in the village, as well as other indicators of village accessibility such as connectivity with a paved road or electricity, access to school and literacy did not improve between 2005 and 2013 for these vulnerable children.

Such findings about children and youth with disabilities are at odds with overall progress in access to school and basic learning outcomes for children in Afghanistan in general [85]. A growing literature indicates that vulnerable children, particularly those with disabilities do not usually benefit from similar improvements in access to school in countries going through reconstruction after a conflict or a crisis or more generally in low income countries promoting education [17, 86]. In the specific context of Afghanistan, a cross sectional study already showed that in 2005 access to school and learning basic cognitive skills were more challenging for children with disabilities [32]. Unfortunately, things have not improved significantly since then.

Overall lack of improvement in access to school and basic learning hides differences between children with disabilities according to various sociodemographic characteristics and identities [87]. These differences were not linked to the economic status of the family as shown by the absence of difference according to the asset index. Yet, we know that compared to non-disabled family, such association between family deprivation, disability and lack of access to school has been identified in other conflict or low-income settings [61, 86, 88]. An important factor of increased access and better learning was the fact that the head of household was himself (or herself in few cases) educated. An educated father or mother is more likely to understand that his/her child can learn and should be educated avoiding widespread prejudice–driven by the social stigma associated to disability in many traditional societies- that children with disabilities are unable to learn [86, 89].

Gender is an important factor associated with school access and learning outcome. Girls with disabilities had lower odds of accessing the classroom and learning. In Afghanistan, the dual unfavorable identity of being disabled and a girl is particularly hurting girls with disabilities’ education [90]. In fact, traditional and religious beliefs have consistently discouraged girl’s education in Afghanistan [51]. Existing initiatives to promote girls education such as implementing village-based schools have improved girls’ access [52]. Community initiatives to empower women such as the BRAC’s life skills education and livelihoods trainings for young Afghan women have been also promoted [53]. However, our findings indicate that girls with disabilities have been to date largely left out.

The socially identified cause of disability was another factor influencing school access and learning. Traditional beliefs about disability also explain that children with mental disability and those who were born with a disability were less likely to access schools. Researchers have shown that disability acquired at birth or from an unknown cause–called *Mayub* -or having a mental or intellectual or learning disability–called *Dewana* (both being derogatory terms) are considered by lay beliefs to be associated to a supernatural cause such as God’s will, fate (*kismet*), Djins or the result of black magic [91]. Such perceptions not only affect the social status of the person with disabilities herself who is often kept hidden and away from the outside world, but the entire family as well as she bears the blame and the responsibility for the impairment.

Children and youth with disabilities–whatever the cause of the type of disability- reported less mental distress and anxiety in 2013 compared to 2005. This encouraging finding may indicate that stigma traditionally associated to mental disability and particularly to disability without a defined cause [92] might be increasingly challenged in the Afghan society, therefore less likely to be expressed publicly. Eventually, this means that children and youth with disabilities might become less discriminated against and therefore see reduction in the harm that goes with internalization of the prejudice endured or self-stigma [93, 94]. This change might have been driven by multiple communication and awareness campaigns by organizations of persons with disabilities, the Afghan Government, United Nations agencies and non-governmental agencies that aim to sensitize the Afghan population to promote empathy and inclusive attitudes towards persons with disabilities [95]. The change can also be explained by better mental health resilience of those children than in the past, when facing stigma.

Nevertheless, signs of anxiety and distress for children with disabilities decreased over the considered period. But findings do not show that the presence of a school in the village played a significant role. For schools to be truly protective of the psychological wellbeing of all children, including those with functional, emotional or behavioral difficulties—while reducing failure of learning, certain conditions have been shown to be impactful in high income countries: student centered, engaging and conducive learning environment, respectful and trustful interactions between students and between teacher and students, clear expectations [96–101]. In conflict and post conflict contexts, the focus has been on measuring the effectiveness of mental health and psychosocial interventions essentially tested in the classroom [102]. Many studies in conflict and post-conflict countries–in Burundi, Democratic Republic of Congo, Indonesia, Nepal or Sri Lanka—did not find a significant effect of these interventions in reducing symptoms of depression and anxiety or post-traumatic stress disorders [14, 19, 103, 104].

The present study has several limitations. First, we compare education outcomes using two cross-sectional with an eight years gap. We could not follow a cohort of children from grade 1 to 10 for instance to measure dropout rate and rate of basic learning outcomes achievement and protective factor of staying in school at different stages of the education process. Additionally, the statistical power of our estimates is weaker compared to a longitudinal study, as we must account for between subject variations. However, comparing children and youth with disabilities of similar demographic and socio-economic background in the same geographical areas of Afghanistan allows for measuring the impact of investment in school on performance and protection of children and youth with disabilities. In any case, the challenge of following a cohort of children through a long period of time in a post-conflict setting such as Afghanistan may have led to a high level of attrition, diminishing the advantage of retaining the same respondents. Second, mental distress was measured using two questions rather than by a multi-item questionnaire such as the Birleson Depression Self-Rating Scale that has been adapted and validated for Afghanistan [5]. However, preliminary psychometric analyses were conducted to evaluate the comparability of assessments across time. Item response theory (IRT) assessed the latent construct of mental distress within each year. IRT models allowed indicators of mental distress to load freely onto a continuous latent factor (theta) with variances fixed at one and mean of zero (Asparouhov & Muthén, 2016). Item characteristics estimated the difficulty (the severity of the indicator for mental distress) and discrimination (the ability of indicators to demarcate risk) for each item. The estimates as well as plots of the item information distributions were reviewed to identify indicators of risk (one standard deviation away from the latent mean of distress) with reliable discrimination (sharp slopes). In both 2005 and 2013, items that assessed child sadness and anger fit the criteria for strong indicators, despite using different wording. Concordance with qualitative data collected to construct measures raised confidence for validity. The items used to measure mental distress in both surveys were subject to a process of validation. We tested the questions for content validity with local psychologists and through in-depth qualitative interviews with potential respondents to establish if their understanding of the question corresponded to its intended meaning. We prompted respondents with questions to check participants’ understanding of sadness and anger, and to identify terms and expressions used to explain these notions, as well as to ascertain their understanding of the questions to make sure that the items’ meaning was understood. Third, our study examined disability and did not compare persons with disabilities to non-disabled people. Therefore, we can only argue that access did not improve for our particular group within the time span we scrutinized. Fourth, we did not conduct the study in the same villages in 2005 and 2013. We assume that the situation did not worsen between 2005 and 2013 and that if a village had a school, electricity and a paved road, this village still had them in 2013. This is a rather strong assumption as we know Taliban have been closing schools in some provinces. But at the time of the survey, in 2013, this was hardly the case in the provinces where the study took place. Such issues of forbidding girls to go to school as well as female teachers are more prevalent in the Southern part of the country than in the Northeastern part. Five, we assessed literacy only through a short text that the child had to read and write. More sophisticated ways of evaluating literacy do exist in the literacy development literature including in Afghanistan [105]. Yet, the enumerator had a follow up question to check if the child understood what he was reading and writing. Finally, security conditions deteriorated considerably between the two periods and we had to exclude some villages under Taliban control from our sample in 2013 potentially overestimating the impact of investment in education.

## Conclusion and recommendation

There is a lack of evidence about what intervention might improve the child learning experience and wellbeing in LICS. This is even more true for what might improve the learning experience of children with disabilities and in conflict settings. new initiatives are needed to investigate this important topic.

Existing interventions in LICS (e.g., class size, teacher incentives, school management accountability, cash transfer) have shown little effect on cognitive skills and none investigated mental wellbeing or non-cognitive skills [106, 107]. For instance, a study in Kenya showed that providing textbooks improved test scores amongst the top 20% of students only, due to the difficult level of the content [108]. Building “girl friendly” schools [52, 109], supporting low performing children with adapted remedial instruction interventions [86, 110, 111] have been shown to increase standardized test scores, but no study has measured the impact of interventions that specifically promote quality education in LICs, particularly in conflict contexts [112]. Existing school interventions have shown mixed results in terms of improving child mental wellbeing in conflicts settings [14, 20, 104]. Lack of studies is even more alarming in the field of disability and inclusive education.

Guidelines aiming at including children with disabilities have been elaborated by the Inter-agency Network on Education in Emergencies (INEE) [31]. Recommendations include: physically accessible buildings; training and support to teachers using local resources and technology; pedagogy that combine and balance classroom integration and learning; peer support to children with disabilities; and fighting prejudice and discrimination amongst teachers, parents, other children, communities, humanitarian actors and policy makers.

Available evidence points in the direction of a multilevel multipronged adaptation of the existing system to improve the learning experience and promote children’s resilience, particularly for the most vulnerable ones, such as children with disabilities in conflict context. At the family level, sensitization about the child with disability’s capacity to learn as well as encouragement to send her or him to school as a way to break the cycle of isolation and exclusion -a chronic stressor that fuel low self-esteem and mental distress and anxiety- entail to challenge the Afghan traditional stigma of disability. This will require a concerted effort to intervene at the community level to break the vicious cycle of attitudes, beliefs, prejudice and discrimination towards persons with disabilities in general and children with disabilities in particular based on cause and type [91, 92]. Awareness and sensitization also have the potential to reduce the risk of violence and bullying [95]. More importantly, at the school level, promoting a conducive learning environment in which the teacher is valorized by good working conditions and extensive training to be able to address the various needs of children might contribute to make school an undisputable protective environment for all children to flourish and be resilient. In particular, future research will need to explore processes of implementation and outcomes of participatory interventions engaging children, peers, parents, teachers, community members concurrently at these various multiple ecological levels in promoting inclusion and building child resilience.
